# Standardized norm data for three self-report scales on egocentric and allocentric environmental spatial strategies

**DOI:** 10.1016/j.dib.2016.06.039

**Published:** 2016-06-29

**Authors:** Stefan Münzer, Benedict C.O.F. Fehringer, Tim Kühl

**Affiliations:** University of Mannheim, Mannheim, Germany

**Keywords:** Environmental spatial strategies, Individual differences, Self-reported spatial ability, Standardized norms

## Abstract

Standardized norm data for three scales of a 19-item self-report measure on environmental spatial strategies are provided. This self-report measure comprises egocentric spatial strategies, an allocentric mental map strategy and knowledge of cardinal directions as three separate scales, “Validation of a 3-factor structure of spatial strategies and relations to possession and usage of navigational aids” (Münzer et al., 2016) [3]. The data are based on a large sample (*N*>4000) representative for the population in Germany. Standardized norm data for men and women in different age groups are provided through percentile ranks and *T*-values.

**Specifications Table**

**Table:** 

Subject area	Psychology
More specific subject area	Environmental spatial cognition, spatial learning, orientation, individual differences, measurement
Type of data	Tables
How data was acquired	Survey; 3-factor 19-item Questionnaire on Spatial Strategies FRS
Data format	Raw, calculated
Experimental factors	Data were separated with respect to sex and age group
Experimental features	Raw data, percentile ranks, *T*-values
Data source location	Germany
Data accessibility	Data are within this article

**Value of the data**

• Standardized norm data based on a large, representative sample helps assessing measured individual differences in self-reported environmental abilities
• Standardized norm data based on a large, representative sample helps assessing measured self-reported environmental abilities in particular samples (e.g., student samples, patient samples)
• Separation of standardized norm data by sex and different age groups helps to assess individual differences with respect to relevant reference groups and age cohorts.

## 1 Data

Standardized norm data for each of the three scales of a 19-item self-report measure on environmental spatial strategies are provided based on a representative sample (*N*>4000). Standardized norm data for men and women and for different age groups from <30 years to 50–80 years are separately provided – see Table A1, Table A2, Table A3, Table A4, Table A5, Table A6, Table A7 with this article. Standardized norm data comprise the percentile rank and two *T*-values (*T*-values based on means and standard deviations conserving the interval scale information, and *T*-values corresponding to the percentile ranks). Data were collected through the GESIS panel [5], and these data are generally accessible for scientific purposes (http://www.gesis.org/en/services/data-collection/gesis-panel/gesis-panel-data-usage/).

## 2 Experimental design, materials and methods

### 2.1 Data resource

Participants were panelists of the GESIS panel, a sample encompassing the German speaking population permanently residing in Germany. For recruitment, a random sample was drawn from municipal population registers, and panelists were interviewed in personal house visits. The omnibus survey waves started in February 2014. Since then, data have been collected on a bi-monthly basis. At the beginning of the panel survey, about 65% of the 4900 panelists participated online (with web-based surveys) and about 35% of the panelists participated offline (mail questionnaires are sent to those participants who are not able or do not want to participate online). Every interviewed person was incentivized for the recruitment interview. Likewise, participants are incentivized for the data collection waves. The present data collection was integrated in the GESIS panel data collection wave "ba" (version number ZA5665, study code "ag").

Data were collected between February and April 2014 from 2223 female and 2057 male participants. The web-based online survey was answered by 2790 participants. Another 824 participants answered an offline, paper-based questionnaire with the same items. For another 666 participants, information about the online/offline format was not provided.

### 2.2 Sex, age groups, nationality and missing data

In 243 cases, missing values occurred in some individual items of the 19-item German Questionnaire on Spatial Strategies [4]. These data were analyzed for *missing completely at random* (MCAR) using the non-parametric test of the MissMech-*R*-package [1]. There was no evidence to reject the MCAR hypothesis. After exclusion of these cases, the data set consisted of 4037 participants (2091 female, 1946 male). 2621 participants filled out the online form and 792 participants an offline form (for 624 cases, there was no information about the online vs. the offline format.) The age was provided in categories (756 participants were younger than 30 years; 602 participants were between 30 and 39 years old; 936 participants were between 40 and 49 years old; 916 participants were between 50 and 59 years old, and 815 participants were between 50 and 80 years old). Of the 4037 participants, 3852 had German nationality.

Calculating norm data for female and male participants and different age groups required to separate the data with respect to sex and age group. For these analyses, another twelve participants were excluded because of missing age data. The MCAR analysis showed no systemic changes by deleting these cases from the original data set. Accordingly, 4025 participants (2085 females and 1940 males) were included in the analyses.

### 2.3 Spatial strategy scales and items on use and possession of navigational aids

Participants answered the 19 items of the Questionnaire on Spatial Strategies [4] as well as eight additional items asking for possession and use of navigational aids such as GPS-based navigation assistance systems and maps.

The first two factors of the self-report measure FRS reflect the distinction between an egocentric (Factor I) and an allocentric (Factor II) spatial reference frame. Corresponding to its core meaning, the item indicating self-reported sense of direction loaded on the egocentric factor, but not on the allocentric factor. The egocentric strategies involved both directional strategies and route-based strategies. In addition, knowledge about cardinal directions was found to be a separate factor (Factor III). The factor structure was confirmed using confirmatory factor analyses [3].

Participants also answered questions included in further studies that were carried out within the same data collection wave. GESIS provides reports and study descriptions on these studies (http://www.gesis.org/en/services/data-collection/gesis-panel/gesis-panel-data-usage/). Each survey wave has about twenty minutes interviewing time and consists of two parts. About fifteen minutes of survey time are reserved for submitted studies (such as the present study). The second part of each survey wave (about five minutes of interviewing time) is reserved for longitudinal core study topics developed by GESIS.

### 2.4 Calculation of standardized norm data

For each of the three scales of the FRS, the percentile rank and two *T*-values were computed, for the complete sample as well as for subsamples separated with respect to sex (women and men) and age groups. The first *T*-values were computed based on means and standard deviations conserving the interval scale information. The second *T*-values were computed according to McCall [2] and corresponded to the percentile ranks. This ordinal transformation conserves the theoretical *T*-value distribution (e.g., 68.2% participants have *T*-values between 40 and 60).

The standardized norm tables for the three scales are provided in Table A1, Table A2, Table A3, Table A4, Table A5, Table A6, Table A7. Table A7 is the conversion table between the percentile ranks and the *T*-value according to McCall [2].

A raw value of 43 for the global/egocentric (SOD) strategy scale means that 47% (Table A2, columns 6 and 9) of the women have a score of 43 or lower on this scale. The corresponding *T*-value is 50 (Table A2, column 10), based on the mean and standard deviation of the score distribution. The corresponding McCall-*T*-value is 49 (see Table A7, columns 3 and 4). A larger difference between *T*-values is obtained for a raw value of 17 for men on the global/egocentric (SOD) strategy scale (Pr=1%, Table A2, columns 1 and 2). The *T*-value (Table A2, column 3) is 20, but the McCall-*T*-value is 27 (Table A7, columns 1 and 2).

To compute group statistics, the non-corrected *T*-value is needed. For statements about how many people have a certain raw value or whether a certain person lies in a specific population area, the *T*-value according to McCall [2] is applied.

## Figures and Tables

**Table A1:** Norm table for the global/egocentric (SOD) strategy scale (by age).

Raw	<30 years		30–39 years		40–49 years		50–59 years		60–79 years	
	PR	*T*	PR	*T*	PR	*T*	PR	*T*	PR	*T*
10	0	22	0	20	0	23	0	24	0	23
11	0	23	0	21	0	24	1	25	1	24
12	1	24	0	22	1	25	1	26	1	25
13	1	24	1	22	1	25	1	26	1	25
14	1	25	1	23	1	26	2	27	1	26
15	1	26	2	24	1	27	2	28	2	27
16	1	27	2	25	2	28	3	28	3	28
17	2	27	2	25	2	28	3	29	3	28
18	2	28	3	26	3	29	4	30	4	29
19	3	29	3	27	4	30	5	30	4	30
20	4	30	4	28	4	30	6	31	5	30
21	4	30	4	29	5	31	6	32	6	31
22	5	31	4	29	5	32	7	32	7	32
23	5	32	5	30	7	33	8	33	7	32
24	6	33	6	31	8	33	9	34	8	33
25	7	33	7	32	9	34	10	34	9	34
26	8	34	8	32	10	35	11	35	10	34
27	9	35	8	33	12	35	12	36	11	35
28	10	36	10	34	13	36	13	36	12	36
29	12	36	11	35	14	37	14	37	12	36
30	13	37	12	35	15	38	15	38	13	37
31	14	38	13	36	17	38	16	39	14	38
32	15	39	13	37	18	39	18	39	15	38
33	17	40	15	38	19	40	19	40	17	39
34	19	40	15	39	20	40	20	41	18	40
35	21	41	16	39	21	41	22	41	20	41
36	23	42	18	40	23	42	23	42	21	41
37	24	43	19	41	24	43	25	43	22	42
38	26	43	20	42	26	43	26	43	23	43
39	29	44	22	42	28	44	28	44	25	43
40	31	45	23	43	30	45	29	45	26	44
41	33	46	24	44	32	45	31	45	28	45
42	35	46	26	45	34	46	33	46	30	45
43	38	47	28	45	36	47	35	47	32	46
44	40	48	30	46	38	48	36	47	34	47
45	44	49	31	47	39	48	39	48	36	47
46	45	49	35	48	41	49	41	49	38	48
47	49	50	38	49	44	50	43	50	40	49
48	51	51	40	49	46	50	45	50	43	49
49	54	52	41	50	49	51	47	51	46	50
50	56	52	46	51	52	52	50	52	47	51
51	59	53	49	52	54	53	52	52	50	51
52	62	54	53	52	56	53	55	53	52	52
53	65	55	58	53	59	54	57	54	55	53
54	68	55	62	54	62	55	61	54	58	54
55	71	56	64	55	65	55	64	55	61	54
56	74	57	68	55	68	56	66	56	63	55
57	77	58	73	56	71	57	70	56	68	56
58	80	58	78	57	74	58	74	57	71	56
59	82	59	79	58	78	58	77	58	73	57
60	84	60	83	59	81	59	80	58	76	58
61	86	61	86	59	84	60	83	59	79	58
62	87	62	89	60	86	60	85	60	81	59
63	90	62	90	61	89	61	87	61	83	60
64	92	63	92	62	91	62	89	61	85	60
65	94	64	93	62	93	62	91	62	88	61
66	95	65	95	63	95	63	93	63	90	62
67	97	65	96	64	97	64	95	63	92	62
68	98	66	97	65	97	65	97	64	94	63
69	99	67	98	65	99	65	98	65	96	64
70	100	68	100	66	100	66	100	65	100	64

**Table A2:** Norm table for the global/egocentric (SOD) strategy scale (by sex).

Raw	Male		Female		Raw	Male		Female		Raw	Male		Female	
	Pr	*T*	Pr	*T*		Pr	*T*	Pr	*T*		Pr	*T*	Pr	*T*
10	0	14	0	27	37	12	37	34	45	64	86	60	94	64
11	0	15	1	28	38	13	38	36	46	65	88	61	95	65
12	0	15	1	28	39	14	38	38	47	66	91	62	96	65
13	0	16	2	29	40	16	39	41	48	67	93	62	97	66
14	0	17	2	30	41	17	40	43	48	68	95	63	98	67
15	0	18	3	30	42	18	41	45	49	69	97	64	99	67
16	0	19	4	31	43	20	42	47	50	70	100	65	100	68
17	1	20	5	32	44	22	43	49	50					
18	1	21	6	33	45	24	44	51	51					
19	1	21	7	33	46	26	44	54	52					
20	1	22	8	34	47	28	45	56	52					
21	2	23	8	35	48	31	46	58	53					
22	2	24	10	35	49	34	47	61	54					
23	2	25	11	36	50	37	48	63	54					
24	3	26	12	37	51	40	49	65	55					
25	3	26	14	37	52	43	50	67	56					
26	3	27	16	38	53	46	50	71	56					
27	4	28	17	39	54	50	51	73	57					
28	4	29	19	39	55	53	52	76	58					
29	5	30	20	40	56	57	53	78	58					
30	6	31	21	41	57	62	54	81	59					
31	7	32	23	41	58	66	55	83	60					
32	7	32	25	42	59	70	56	85	61					
33	8	33	27	43	60	74	56	87	61					
34	9	34	28	43	61	77	57	89	62					
35	10	35	30	44	62	80	58	90	63					
36	11	36	32	45	63	83	59	92	63					

**Table A3:** Norm table for the allocentric “mental map” strategy scale (by age).

Raw	<30 years		30–39 years		40–49 years		50–59 years		60–79 years	
	PR	*T*	PR	*T*	PR	*T*	PR	*T*	PR	*T*
7	1	30	1	29	2	30	1	31	1	29
8	3	31	2	30	3	31	3	31	2	30
9	3	32	3	31	4	32	5	32	2	31
10	4	33	5	32	6	33	6	33	3	32
11	5	34	6	33	7	34	7	34	6	33
12	8	35	8	34	8	35	10	35	8	34
13	10	36	10	35	10	36	12	36	9	35
14	12	37	13	36	12	37	15	37	10	35
15	13	38	14	37	15	38	16	38	12	36
16	15	39	16	38	17	39	18	39	14	37
17	18	40	17	39	20	40	20	40	16	38
18	21	41	19	40	23	41	22	40	18	39
19	24	42	22	41	25	41	25	41	20	40
20	28	43	24	42	28	42	27	42	22	41
21	32	44	27	43	31	43	29	43	25	42
22	36	45	29	44	33	44	31	44	27	43
23	38	46	32	45	36	45	34	45	30	44
24	42	47	34	45	39	46	37	46	32	45
25	46	48	36	46	41	47	39	47	35	45
26	49	49	39	47	43	48	42	48	38	46
27	53	50	43	48	46	49	45	49	40	47
28	56	51	46	49	49	50	49	50	44	48
29	60	52	50	50	53	51	51	50	47	49
30	62	53	53	51	56	52	54	51	50	50
31	65	54	56	52	59	53	57	52	52	51
32	68	55	60	53	62	53	60	53	55	52
33	72	56	64	54	65	54	62	54	58	53
34	75	57	69	55	67	55	66	55	62	54
35	78	58	73	56	71	56	69	56	65	54
36	80	59	76	57	74	57	72	57	67	55
37	83	60	78	58	77	58	74	58	70	56
38	86	61	81	59	80	59	77	59	73	57
39	87	62	83	60	82	60	80	59	76	58
40	90	63	85	61	85	61	83	60	80	59
41	92	64	88	62	87	62	86	61	82	60
42	94	65	90	63	90	63	90	62	85	61
43	95	66	92	64	92	64	92	63	88	62
44	96	67	94	65	94	65	94	64	89	63
45	97	68	95	66	95	65	96	65	91	63
46	98	69	97	67	97	66	97	66	93	64
47	98	70	98	67	97	67	98	67	95	65
48	99	71	99	68	99	68	98	68	97	66
49	100	72	100	69	100	69	100	69	100	67

**Table A4:** Norm table for the allocentric “mental map” strategy scale (by sex).

Raw	Male		Female		Raw	Male		Female	
	Pr	*T*	Pr	*T*		Pr	*T*	Pr	*T*
7	1	25	2	32	34	57	52	78	58
8	1	26	3	33	35	61	53	81	59
9	2	27	5	34	36	64	54	83	60
10	2	28	7	35	37	67	55	85	61
11	3	29	10	36	38	71	56	87	62
12	4	30	13	37	39	74	57	88	63
13	5	31	15	38	40	78	58	90	64
14	6	32	18	39	41	81	59	92	65
15	7	33	21	40	42	85	60	94	66
16	9	34	23	41	43	88	61	95	67
17	10	35	26	42	44	90	62	96	68
18	12	36	29	43	45	92	63	97	68
19	14	37	33	44	46	95	64	98	69
20	15	38	36	45	47	96	65	98	70
21	18	39	40	46	48	98	66	99	71
22	20	40	43	47	49	100	67	100	72
23	21	41	46	48					
24	24	42	49	49					
25	27	43	52	50					
26	30	44	54	50					
27	33	45	57	51					
28	36	46	61	52					
29	40	47	64	53					
30	43	48	66	54					
31	45	49	69	55					
32	49	50	72	56					
33	53	51	75	57					

**Table A5:** Norm table for the cardinal directions strategy scale (by age).

Raw	<30 years		30–39 years		40–49 years		50–59 years		60–79 years	
	PR	*T*	PR	*T*	PR	*T*	PR	*T*	PR	*T*
2	20	38	12	34	12	34	10	32	6	30
3	27	41	15	37	15	37	13	35	10	33
4	40	43	25	40	24	39	19	38	15	36
5	46	46	29	42	28	42	23	40	19	38
6	55	49	39	45	37	45	30	43	25	41
7	61	51	46	48	43	47	35	45	30	44
8	69	54	54	50	54	50	44	48	40	46
9	73	57	61	53	60	53	50	51	46	49
10	81	59	73	56	71	55	62	53	55	52
11	86	62	79	59	77	58	69	56	61	54
12	93	64	90	61	88	61	83	59	77	57
13	95	67	94	64	92	63	88	61	83	60
14	100	70	100	67	100	66	100	64	100	63

**Table A6:** Norm table for the cardinal directions strategy scale (by sex).

Raw	Male		Female	
	Pr	*T*	Pr	*T*
2	6	30	17	37
3	8	32	23	39
4	14	35	34	42
5	17	38	39	45
6	24	41	48	47
7	29	44	54	50
8	39	46	63	53
9	45	49	69	55
10	56	52	79	58
11	64	55	84	61
12	79	57	92	63
13	86	60	94	66
14	100	63	100	69

**Table A7:** Conversion table between percentile rank and *T*-value according to McCall [2].

PR	*T*	PR	*T*	PR	*T*
0	–	34	46	68	55
1	27	35	46	69	55
2	29	36	46	70	55
3	31	37	47	71	56
4	32	38	47	72	56
5	34	39	47	73	56
6	34	40	47	74	56
7	35	41	48	75	57
8	36	42	48	76	57
9	37	43	48	77	57
10	37	44	48	78	58
11	38	45	49	79	58
12	38	46	49	80	58
13	39	47	49	81	59
14	39	48	49	82	59
15	40	49	50	83	60
16	40	50	50	84	60
17	40	51	50	85	60
18	41	52	51	86	61
19	41	53	51	87	61
20	42	54	51	88	62
21	42	55	51	89	62
22	42	56	52	90	63
23	43	57	52	91	63
24	43	58	52	92	64
25	43	59	52	93	65
26	44	60	53	94	66
27	44	61	53	95	66
28	44	62	53	96	68
29	44	63	53	97	69
30	45	64	54	98	71
31	45	65	54	99	73
32	45	66	54	100	–
33	46	67	54		

## Appendix A

See Table A1, Table A2, Table A3, Table A4, Table A5, Table A6, Table A7 here.
